# Lack of Knowledge and Training About Antibiotic Resistance Among Community Pharmacists in Bangladesh: A Cross‐Sectional Study

**DOI:** 10.1002/hsr2.72866

**Published:** 2026-07-25

**Authors:** Md. Jubayer Hossain, Musab Shahariar, Lamia Hasan Joarder Barsha, Muhibullah Shahjahan, Syeda Tasneem Towhid, Md. Kamrul Sheikh, Md. Mehedi Hasan, Md. Habibur Rahaman, Md. Shahnewaz Sazid

**Affiliations:** ^1^ Center for Health Innovation, Research, Action, and Learning – Bangladesh (CHIRAL Bangladesh) Dhaka Bangladesh; ^2^ Population Health Studies Division Center for Health Innovation, Research, Action, and Learning – Bangladesh (CHIRAL Bangladesh) Dhaka Bangladesh; ^3^ Department of Pharmacy Islamic University Kushtia Bangladesh; ^4^ Department of Microbiology Jagannath University Dhaka Bangladesh; ^5^ Department of Biotechnology and Genetic Engineering Islamic University Kushtia Bangladesh

**Keywords:** antibiotic misuse, antibiotic resistance, antibiotic stewardship, Bangladesh, community pharmacists

## Abstract

**Background and Aims:**

Antibiotic resistance poses a critical global public health threat, disproportionately affecting low‐ and middle‐income countries. Community pharmacists in Bangladesh serve as key healthcare intermediaries, yet significant knowledge gaps regarding antibiotic use and resistance persist. This study assessed the level of knowledge and training among community pharmacists in Bangladesh regarding antibiotic resistance and explored their relationship with antibiotic dispensing practices.

**Methods:**

A cross‐sectional study was conducted among community pharmacists in Bangladesh using a validated self‐administered questionnaire covering demographic characteristics, knowledge and attitudes towards antibiotic use and resistance, and dispensing practices.

**Results:**

Among 191 participants, only 8.4% demonstrated adequate knowledge of antibiotic resistance and 38% had received antimicrobial stewardship training. Training was strongly associated with more favorable attitudes (*p* < 0.001) and better dispensing practices (*p* < 0.001), but not with overall knowledge levels (*p* = 0.12).

**Conclusion:**

Substantial knowledge and training deficits exist among community pharmacists in Bangladesh. Targeted and continuing education programs are urgently needed to improve antibiotic dispensing practices and reduce antibiotic resistance.

## 1 Introduction

Antibiotic resistance is a critical global health issue that threatens the efficacy of antimicrobial treatments [1, 2]. Widespread and often inappropriate antibiotic use accelerates the development of resistance mechanisms in bacteria, increasing mortality and prolonging hospital stays [3, 4]. Antibiotic‐resistant bacterial infections are estimated to be associated with approximately 5.0 million deaths annually, with 4.3 million occurring in low‐ and middle‐income countries (LMICs) [5].

In Bangladesh, inappropriate antibiotic dispensing is well documented among both qualified and unqualified healthcare providers [6, 7, 8, 9, 10]. Approximately 80% of the population relies on retail drug shops for healthcare [11], and nearly half of all antibiotics are dispensed without a prescription [12]. Bangladesh's Antimicrobial Resistance Surveillance Report (2016–2023) indicates that 73% of antibiotics used belong to the World Health Organization Watch category [13], reflecting a broader pattern in lower‐middle‐income countries [14]. Policy responses include the National Antimicrobial Resistance Surveillance Strategy (2020–2025) [15] and the red labeling initiative implemented by the Directorate General of Drug Administration in collaboration with the World Health Organization [16].

Community pharmacists are pivotal gatekeepers in antibiotic dispensing, yet dispensing quality remains suboptimal due to persistent knowledge gaps and deviations from established guidelines [17, 18]. Assessing pharmacist knowledge, training, and practices at the community level is essential to inform targeted interventions and policy enforcement [19, 20, 21, 22].

This study aimed to: (i) assess current knowledge about antibiotic resistance among community pharmacists in Bangladesh; (ii) identify the extent of training received on this topic; and (iii) explore the relationship between knowledge, training, and antibiotic dispensing practices.

## 2 Methods

### 2.1 Study Design and Setting

A cross‐sectional study was conducted among pharmacists working in retail pharmacies across Magura, Kushtia, and Dhaka districts of Bangladesh between January and April 2023.

### 2.2 Study Population and Eligibility

Bangladesh has an estimated 191,512 registered retail pharmacies [23]. Pharmacists are classified as Grade A (bachelor's degree in pharmacy, approximately 21,539), Grade B (3‐year Diploma in Pharmacy, approximately 6217), or Grade C (3‐month dispensing training, approximately 166,531), all regulated by the Pharmacy Council Bangladesh [24, 25]. Pharmacists working in retail drug outlets who agreed to participate were included; other healthcare providers were excluded.

### 2.3 Sample Size and Data Collection

A minimum sample of 384 was estimated (95% confidence interval, 5% margin of error; *n* = *z* ^2^ *pq*/*d* ^2^). Data were collected via a validated paper‐based self‐administered questionnaire distributed in person during working hours; a random sampling method was used. This study was approved by the CHIRAL Bangladesh Ethical Review Committee (Reference: CHIBAN1MAR2023‐0001).

### 2.4 Survey Instrument and Scoring

The questionnaire was developed through an extensive literature review [26, 27] and comprised four sections: demographics (8 items), knowledge (9 items), attitudes towards antimicrobial stewardship (10 items), and practices (10 items). Percentage scores were computed for each domain; scores of 80%–100% indicated good performance and 0%–79% indicated poor performance. The scoring system was adapted from Mufwambi et al. [28], using the same cut‐off values to ensure comparability with previously published research.

### 2.5 Statistical Analysis

Descriptive statistics were computed for all demographic variables. Between‐group differences (trained vs. untrained pharmacists) were assessed using Fisher's exact test and Pearson's chi‐squared test. Univariate and multivariable ordinary least‐squares (OLS) regression analyses identified factors associated with knowledge, attitude, and practice scores. All relevant covariates were entered simultaneously; multicollinearity was assessed using Variance Inflation Factors (threshold = 5). Statistical significance was set at *p* < 0.05. Analyses were conducted in R version 4.3.0 using the gtsummary package [29].

## 3 Results

### 3.1 Characteristics of Study Participants

A total of 296 pharmacists were approached, of whom 191 completed the questionnaire, corresponding to a response rate of 64.5%. Non‐participation was attributable primarily to time constraints during working hours, reluctance to engage with research activities, and absence from the pharmacy at the time of the visit; no systematic differences were identified between participants and non‐participants based on observable characteristics. The largest age group was 20–29 years (33%). Most participants held non‐formal pharmacy qualifications (43% other educational backgrounds; 37% Pharmacy Certificate Registration Course) and 58% were pharmacy owners. The largest experience group was 0–5 years (31%), and 46% dispensed antibiotics very frequently. Only 38% had received formal antimicrobial stewardship training (Table 1).

**Table 1:** Demographic characteristics of study participants (*N* = 191).

Characteristics	*n* (%)
What is your gender?	
Female	5 (2.6%)
Male	186 (97%)
What is your age group? (years)	
20–29	63 (33%)
30–39	57 (30%)
40–49	41 (22%)
50–59	23 (12%)
60 years or above	5 (2.6%)
What is your highest level of education completed?	
No formal education	1 (0.5%)
Pharmacy Certificate Registration Course	70 (36.6%)
Diploma in Pharmacy	16 (8.4%)
B. Pharm	14 (7.3%)
B. Pharm Professional	8 (4.2%)
Others	82 (43%)
What is your current job title?	
Staff pharmacist	51 (27%)
Pharmacy manager	19 (10%)
Pharmacy owner	110 (58%)
Others	9 (4.8%)
How many years of experience do you have as a community pharmacist? (years)	
0–5	59 (31%)
6–10	55 (28.5%)
11–15	30 (15.5%)
16–20	24 (13%)
More than 20 years	23 (12%)
How often do you dispense antibiotics?	
Rarely (less than once a week)	16 (8%)
Occasionally (1–2 times a week)	49 (26%)
Frequently (3–5 times a week)	37 (20%)
Very frequently (more than 5 times a week)	87 (46%)
Have you received training in antimicrobial stewardship?	
Yes	73 (38%)
No	118 (62%)

### 3.2 Knowledge Towards Antimicrobial Resistance

Trained pharmacists showed significantly better responses on key knowledge items compared with untrained pharmacists, including recognizing that dispensing antibiotics without a prescription is illegal (*p* = 0.001), that it contributes to antibiotic resistance (*p* = 0.004), and that self‐medication is a leading cause of resistance (*p* = 0.041) (Table 2).

**Table 2:** Distribution of responses of knowledge‐related questions (yes/no/don't know) among trained and untrained community pharmacists.

Characteristics	Overall *N* = 191^a^	Untrained, *N* = 118^a^	Trained, *N* = 73^a^	*p* value^b^
Do you think that it is legal to dispense antibiotics without a valid prescription in Bangladesh?				**0.001**
Yes	160 (83.7%)	98 (83%)	62 (85%)	
No	12 (6.2%)	3 (2.5%)	9 (12%)	
Don't know	19 (9.9%)	17 (14%)	2 (2.7%)	
Do you think that refusing to dispense antibiotics without prescriptions negatively affect the sales and profits of the pharmacy?				**0.008**
Yes	73 (38%)	35 (30%)	38 (52%)	
No	97 (51%)	69 (58%)	28 (38%)	
Don't know	21 (11%)	14 (12%)	7 (9.6%)	
Do you think dispensing antibiotics without a prescription is contributing to the development of antibiotic resistance?				**0.004**
Yes	128 (67%)	70 (59%)	58 (79%)	
No	42 (22%)	29 (25%)	13 (18%)	
Don't know	21 (11%)	19 (16%)	2 (2.7%)	
Do you think self‐medication is the leading cause of antibiotic resistance?				**0.041**
Yes	127 (66%)	74 (63%)	53 (73%)	
No	50 (26%)	31 (26%)	19 (26%)	
Don't know	14 (7.3%)	13 (11%)	1 (1%)	

*Note: p* value < 0.05 was considered statistically significant. Significant values are in bold.

^a^ *n* (%).

^b^ Fisher's exact test; Pearson's chi‐squared test.

### 3.3 Attitude Towards Antimicrobial Resistance

Agreement with positive attitudes was consistently stronger among trained pharmacists. They were more likely to agree that appropriate antibiotic use can reduce resistance (100% vs. 84%, *p* < 0.001), that antimicrobial stewardship programs can reduce misuse (95% vs. 76%, *p* = 0.002), and that such programs should be incorporated at the community pharmacy level (99% vs. 86%, *p* = 0.006) (Table 3).

**Table 3:** Distribution of responses (agree/neutral/disagree) of attitude‐related questions among trained and untrained community pharmacists.

Characteristics	Overall, *N* = 191^a^	Untrained, *N* = 118^a^	Trained, *N* = 73^a^	*p* value^b^
Appropriate use of antibiotics can reduce problems of antibiotic resistance				**< 0.001**
Agree	172 (90%)	99 (84%)	73 (100%)	
Disagree	4 (2.1%)	4 (3%)	0 (0%)	
Neutral	15 (7.9%)	15 (13%)	0 (0%)	
Antimicrobial stewardship programs can reduce the misuse of antibiotics				**0.002**
Agree	159 (83%)	90 (76%)	69 (95%)	
Disagree	6 (3%)	5 (4.2%)	1 (1%)	
Neutral	26 (14%)	23 (19%)	3 (4%)	
Antimicrobial stewardship programs should be incorporated at the community‐pharmacy level				**0.006**
Agree	173 (91%)	101 (86%)	72 (99%)	
Disagree	4 (2%)	4 (3%)	0 (0%)	
Neutral	14 (7%)	13 (11%)	1 (1%)	

*Note: p* value  < 0.05 was considered statistically significant. Significant values are in bold.

^a^ *n* (%).

^b^ Fisher's exact test; Pearson's chi‐squared test.

### 3.4 Practices Towards Antibiotic Use and Dispensing

Trained pharmacists consistently reported safer dispensing practices. They were more likely to ask patients about drug allergies (75%, *p* = 0.024), concomitant medicines (90%, *p* < 0.001), and to advise on dosage adherence and course completion (97%, *p* = 0.001). Counseling about adverse effects (85%, *p* < 0.001) and collaboration with other health professionals on antimicrobial stewardship (45%, *p* = 0.005) were also significantly more common among trained pharmacists (Table 4).

**Table 4:** Distribution of responses (yes/no) of practice‐related questions among trained and untrained community pharmacists.

Characteristics	Overall, *N* = 191^a^	Untrained, *N* = 118^a^	Trained, *N* = 73^a^	*p* value^b^
Do you ask patients if they have any drug allergies before dispensing antibiotics?				**0.024**
Yes	125 (65%)	70 (59%)	55 (75%)	
No	66 (35%)	48 (41%)	18 (25%)	
Do you ask patients if they are on any other medicines before dispensing antibiotics?				**< 0.001**
Yes	144 (75%)	78 (66%)	66 (90%)	
No	47 (25%)	40 (34%)	7 (9.6%)	
Do you dispense antibiotics on prescriptions with complete clinical information?				**0.040**
Yes	169 (88%)	100 (85%)	69 (95%)	
No	22 (12%)	18 (15%)	4 (5%)	
Do you advise patients to adhere to their antibiotic dosage regimen and its course completion?				**0.001**
Yes	167 (87%)	96 (81%)	71 (97%)	
No	24 (13%)	22 (19%)	2 (2.7%)	
In the past week, have you told patients about the adverse effects of using antimicrobials?				**< 0.001**
Yes	126 (66%)	64 (54%)	62 (85%)	
No	65 (34%)	54 (46%)	11 (15%)	
In the past week, have you given medication consultations to patients who bought antimicrobials at the pharmacy?				**< 0.001**
Yes	136 (71%)	73 (62%)	63 (86%)	
No	55 (29%)	45 (38%)	10 (14%)	
In the past week, have you received patients who took a hospital prescription to buy antimicrobial drugs?				**0.005**
Yes	163 (85%)	94 (80%)	69 (95%)	
No	28 (15%)	24 (20%)	4 (5%)	
Do you collaborate with other health professionals on infection control and antimicrobial stewardship?				**0.005**
Yes	63 (33%)	30 (25%)	33 (45%)	
No	128 (67%)	88 (75%)	40 (55%)	

*Note: p* value < 0.05 was considered statistically significant. Significant values are in bold.

^a^ *n* (%).

^b^ Pearson's chi‐squared test.

### 3.5 Level of Knowledge, Attitudes, and Practices

Only 8.4% of pharmacists demonstrated good overall knowledge, with no statistically significant difference between trained and untrained groups (*p* = 0.12). Training was strongly associated with positive attitudes (95% trained vs. 66% untrained; *p* < 0.001) and good practices (59% trained vs. 33% untrained; *p* < 0.001) (Table 5).

**Table 5:** Level of knowledge, attitude, and practices among trained and untrained community pharmacists.

Characteristics	Overall, *N* = 191^a^	Untrained, *N* = 118^a^	Trained, *N* = 73^a^	*p* value^b^
Knowledge level				0.12
Good	16 (8.4%)	7 (5.9%)	9 (12%)	
Poor	175 (92%)	111 (94%)	64 (88%)	
Attitude level				**< 0.001**
Negative	44 (23%)	40 (34%)	4 (5.5%)	
Positive	147 (77%)	78 (66%)	69 (95%)	
Practice level				**< 0.001**
Good	82 (43%)	39 (33%)	43 (59%)	
Poor	109 (57%)	79 (67%)	30 (41%)	

*Note: p* value < 0.05 was considered statistically significant. Significant values are in bold.

^a^ *n* (%).

^b^ Pearson's chi‐squared test.

### 3.6 Regression Analyses

In univariate analyses, older age, longer experience, senior professional roles, and prior antimicrobial stewardship training were positively associated with higher attitude and practice scores (Table 6). Antimicrobial stewardship training was significantly associated with higher knowledge (*β* = 0.56, 95% CI 0.08–1.0, *p* = 0.024), attitude (*β* = 1.1, 95% CI 0.61–1.6, *p* < 0.001), and practice scores (*β* = 1.1, 95% CI 0.68–1.6, *p* < 0.001).

**Table 6:** Univariate analysis of covariates associated with knowledge, attitudes, and practices scores among community pharmacists.

	Knowledge			Attitude			Practice		
Characteristics	Beta	95% CI^1^	*p* value	Beta	95% CI^1^	*p* value	Beta	95% CI^1^	*p* value
Gender									
Female	—	—		—	—		—	—	
Male	0.35	−1.1, 1.8	0.64	2.6	1.1, 4.1	**0.001**	1.9	0.43, 3.3	**0.011**
Age (years)									
20–29	—	—		—	—		—	—	
30–39	0.39	−0.21, 0.99	0.20	0.31	−0.31, 0.92	0.33	0.93	0.37, 1.5	**0.001**
40–49	0.46	−0.20, 1.1	0.17	0.80	0.12, 1.5	**0.021**	1.0	0.39, 1.6	**0.002**
50–59	0.52	−0.28, 1.3	0.21	0.83	0.01, 1.7	**0.048**	1.2	0.42, 1.9	**0.003**
60 or above	1.2	−0.35, 2.7	0.13	1.7	0.13, 3.3	**0.036**	1.9	0.44, 3.3	**0.011**
Education									
B. Pharm	—	—		—	—		—	—	
Diploma in pharmacy	−0.54	−1.8, 0.66	0.38	0.46	−0.77, 1.7	0.46	1.2	0.09, 2.4	**0.036**
Others	−0.15	−1.1, 0.80	0.76	0.26	−0.71, 1.2	0.60	1.3	0.41, 2.2	**0.005**
Pharmacy Certificate Registration Course	−0.49	−1.5, 0.48	0.32	1.0	0.05, 2.0	**0.040**	1.5	0.54, 2.4	**0.002**
Role									
Other	—	—		—	—		—	—	
Pharmacy manager	1.3	−0.06, 2.6	0.062	1.4	0.04, 2.7	**0.044**	0.32	−0.94, 1.6	0.62
Pharmacy owner	1.6	0.51, 2.8	**0.005**	2.5	1.4, 3.6	**< 0.001**	1.2	0.10, 2.2	**0.033**
Staff pharmacist	1.3	0.13, 2.5	**0.031**	1.8	0.60, 3.0	**0.003**	0.29	−0.82, 1.4	0.61
Experience (years)									
0–5	—	—		—	—		—	—	
11–15	0.46	−0.26, 1.2	0.21	0.27	−0.50, 1.0	0.50	1.0	0.30, 1.7	**0.006**
16–20	0.90	0.11, 1.7	**0.026**	0.66	−0.17, 1.5	0.12	1.1	0.35, 1.9	**0.005**
More than 20	0.99	0.19, 1.8	**0.016**	0.73	−0.11, 1.6	0.091	1.0	0.27, 1.8	**0.009**
Dispense antibiotics									
Frequently (3–5 times a week)	—	—		—	—		—	—	
Very frequently (more than 5 times a week)	0.22	−0.43, 0.87	0.50	0.80	0.13, 1.5	**0.020**	0.18	−0.44, 0.81	0.57
Have you received training in antimicrobial stewardship?									
No	—	—		—	—		—	—	
Yes	0.56	0.08, 1.0	**0.024**	1.1	0.61, 1.6	**< 0.001**	1.1	0.68, 1.6	**< 0.001**

*Note: p* value < 0.05 was considered statistically significant. Significant values are in bold.

In multivariable analysis, training remained significantly associated with attitude (*β* = 0.90, 95% CI 0.40–1.4, *p* < 0.001) and practice (β = 0.90, 95% CI 0.43–1.4, *p* < 0.001), but not with knowledge (β = 0.46, *p* = 0.090). Pharmacy owners and staff pharmacists showed significantly higher knowledge and attitude scores compared with other roles. No multicollinearity concerns were identified (GVIF^1^^/^^2D^F range: 1.07–1.16) (Table 7).

**Table 7:** Multivariate analysis of covariates associated with knowledge, attitudes, and practices scores among community pharmacists.

	Knowledge			Attitude			Practice		
Characteristic	Beta	95% CI	*p* value	Beta	95% CI	*p* value	Beta	95% CI	*p* value
Education									
B. Pharm	—	—		—	—		—	—	
Others	−0.28	−1.3, 0.75	0.59	0.15	−0.82, 1.1	0.77	0.96	0.05, 1.9	**0.039**
Role									
Other	—	—		—	—		—	—	
Pharmacy owner	1.6	0.23, 3.0	**0.023**	1.9	0.57, 3.2	**0.005**	0.11	−1.1, 1.3	0.86
Staff pharmacist	1.6	0.19, 2.9	**0.026**	1.5	0.24, 2.8	**0.020**	0.14	−1.1, 1.3	0.82
Have you received training in antimicrobial stewardship?									
No	—	—		—	—		—	—	
Yes	0.46	−0.07, 1.0	0.090	0.90	0.40, 1.4	**< 0.001**	0.90	0.43, 1.4	**< 0.001**

*Note: p* value < 0.05 was considered statistically significant. Significant values are in bold.

Abbreviation: CI, confidence interval.

## 4 Discussion

This study demonstrates that antimicrobial stewardship training is strongly associated with more favorable attitudes and better dispensing practices among community pharmacists in Bangladesh, despite pervasive knowledge deficits in both trained and untrained groups. Only 8.4% of pharmacists showed good overall knowledge of antibiotic resistance, yet training was significantly associated with improved attitude and practice scores. These findings highlight that even short‐term training interventions can meaningfully improve pharmacist behavior, and underscore the urgent need to scale up stewardship programs within Bangladesh's community pharmacy sector.

The predominantly low educational background of our sample, most holding secondary‐level qualifications or a Pharmacy Certificate Registration Course, reflects the regulatory context in Bangladesh, where Grade C pharmacists certified through a 12‐week training operate the majority of retail pharmacies [30, 31]. This structural gap likely explains the wide divergence between knowledge and practice and reinforces the need for structured continuing education targeting the specific competency gaps of Grade C pharmacists.

The significant association between training and attitudes aligns with evidence from Australia, the United Kingdom, and the United States, where stewardship training in community pharmacy has been shown to improve guideline adherence and pharmacist–patient communication [32, 33, 34, 35, 36]. Similarly, barriers identified in our study, including limited training, economic pressures, and attitudes toward non‐prescription dispensing, mirror those reported in Australian community pharmacies [37, 38, 39, 40]. Both trained and untrained pharmacists engaged in non‐prescription antibiotic dispensing, consistent with findings from Spain and Nepal [41, 42], confirming that training alone is insufficient without parallel regulatory enforcement.

The strong association between training and practice is corroborated by studies from Thailand [43, 44], Nepal [45], and Zambia [46], all of which found that education and training significantly influence community pharmacist behavior. Demographic factors including professional role and experience also contributed positively to performance, consistent with observations from Scotland and the United States [47, 48].

This study has limitations. The sample was drawn from three districts and may not fully represent Bangladesh's diverse pharmacy workforce. The cross‐sectional design precludes causal inference, and self‐reported data are susceptible to social desirability bias. Future research should use larger, geographically diverse samples, objective dispensing audits, and longitudinal designs to evaluate the sustained impact of stewardship training.

## 5 Conclusion

This study identifies critical knowledge and training deficits among community pharmacists in Bangladesh, with antimicrobial stewardship training significantly associated with more positive attitudes and better dispensing practices. Targeted, continuing education programs tailored to the specific needs of community pharmacists, particularly Grade C pharmacists are urgently needed to promote responsible antibiotic dispensing and mitigate antibiotic resistance.

## Author Contributions

**Md. Jubayer Hossain:** conceptualization, investigation, writing – original draft, methodology, validation, visualization, writing – review and editing, software, formal analysis, project administration, data curation, supervision, resources. **Musab Shahariar:** writing – original draft, writing – review and editing, validation, data curation. **Lamia Hasan Joarder Barsha:** writing – original draft, writing – review and editing, validation, data curation. **Muhibullah Shahjahan:** writing – original draft, investigation, validation, methodology, writing – review and editing, software, data curation, formal analysis. **Syeda Tasneem Towhid:** project administration, supervision, resources, writing – review and editing, writing – original draft, validation, investigation. **Md. Kamrul Sheikh:** writing – review and editing, writing – original draft, validation, data curation. **Md. Mehedi Hasan:** writing – original draft, writing – review and editing, data curation, validation. **Md. Habibur Rahaman:** writing – original draft, writing – review and editing, validation, data curation. **Md. Shahnewaz Sazid:** writing – original draft, writing – review and editing, validation, data curation.

## Funding

The authors have nothing to report.

## Ethics Statement

The study received ethical approval from the CHIRAL Bangladesh Ethical Review Committee (Reference: CHIBAN1MAR2023‐0001).

## Consent

Informed consent was obtained from all participants prior to data collection.

## Conflicts of Interest

The authors declare no conflicts of interest.

## Artificial Intelligence Disclosure

Artificial intelligence (AI)‐assisted tools were used during the preparation of this manuscript solely for language editing, grammar refinement, and improvement of clarity and readability. All scientific content, data collection, statistical analyses, interpretation of findings, and intellectual contributions were performed exclusively by the authors. The AI tool did not generate, analyses, or interpret any research data, nor did it contribute to the study design or conclusions. The authors take full responsibility for the accuracy, integrity, and originality of the work presented.

## Transparency Statement

Md. Jubayer Hossain, as the lead author, affirms that this manuscript represents an honest, accurate, and transparent account of the study as designed and conducted. No important aspects of the study have been omitted. Any deviations from the original study protocol are disclosed and explained within the manuscript.

## Data Availability

The datasets used and/or analyzed during the current study are available from the corresponding author upon reasonable request. The study data are available from the corresponding author upon reasonable request, subject to compliance with applicable ethical and privacy regulations.
